# Thickness of the hard palate: A new criterion for the diagnosis of osteoporosis?

**DOI:** 10.1007/s00117-024-01318-9

**Published:** 2024-06-06

**Authors:** Daniela Kildal, Tobias Riether, Tilmann Blasenbrey, Dritan Turhani, Gerald Antoch, Meinrad Beer, Margrit-Ann Geibel

**Affiliations:** 1 Upper Valais Hospital Center Visp (SZO), Radiology, Hôpital du Valais, 3930 Visp, Switzerland; 2 Diagnostic and Interventional Radiology, University Hospital Ulm, 89070 Ulm, Germany; 3 Spitalzentrum Oberwallis, Klinik Radiologie, Pflanzettastrasse 8, 3930 Visp, Switzerland; 4 Dento- and Maxillofacial Surgery, University Hospital Ulm, 89070 Ulm, Germany; 5 Dental radiology, University Hospital Ulm, 89070 Ulm, Germany; 6 Center for Oral and Maxillofacial Surgery, Danbue University, 3500 Krems an der Donau, Austria; 7 Institute for Diagnostic and Interventional Radiology, University Hospital Düsseldorf, 40225 Düsseldorf, Germany; 8 Department of Dentistry, Danbue University, 3500 Krems an der Donau, Austria

**Keywords:** Computed tomography, Osteoporosis, Osteopenia, Osteopetrosis, Incidental findings, Computertomographie, Osteoporose, Osteopenie, Osteopetrose, Zufallsbefund

## Abstract

**Background:**

We were looking for an osteoporosis screening in computed tomography (CT) exams, simple and without additional examinations. We hypothesized that the criterion of “decreasing cortical thickness”, may have an influence on the hard palate. Therefore, we investigated whether thickness of the hard palate (HPT) may serve as an indicator of osteoporosis for patients imaged for other reasons.

**Methods:**

Patients with dual-energy x-ray absorptiometry (DXA) and CT were identified by a radiology information system (RIS)-based, full-text search. Measurement of thickness of hard palate done in existing CT image by radiologist and dentist and compared with available findings and DXA measurements.

**Results:**

We identified a “test group”: 57 patients with DXA and CT available out of 449 patient population and we selected further 70 patients without bone diseases as “control groups”. The measurements showed that HPT correlated with age and bone density. The mean HPT was 2.4 mm in normal, 0.9 mm in osteopenia, 0.8 mm in osteoporosis and 5.3 mm in osteopetrosis case. No bone “healthy” patient fell below 1 mm. The relationship between bone density and HPT has not been described previously. HPT was highest in the bone-healthy group and decreased with age, osteopenia, and osteoporosis. Osteopetrosis, as a disease with increased bone density showed an increase in HPT.

**Conclusions:**

HPT correlates with bone disease. We propose a new criterion for assessment on CT and digital volume tomography (DVT) or cone beam computed tomography (CBCT). A threshold of 1.0 mm when applying a simple measurement of HPT on Head CT or DVT may serve as an indicator for potential osteopenia or osteoporosis as incidental finding without extra imaging further diagnosis and treatment leading to early notice of Osteoporosis.

## Abstract (de)

**Hintergrund:**

Wir suchten nach einem einfach reproduzierbaren Osteoporose-Merkmal im Kiefer. Wir stellten die Hypothese auf, dass das Kriterium der „abnehmenden kortikalen Kortikalisdicke“ auf das Palatum durum (PD) anzuwenden ist und prüften den Höhendurchmesser des PD (HPD) als Indikator für Osteoporose.

**Methoden:**

Mit Volltextrecherche im Radiologie-Informationssystem (RIS) identifizierten wir Patienten mit Dual-Energy-Röntgenabsorptiometrie (DXA) und CT (Computertomographie) des Schädels. Die Messung des HPD erfolgte durch Radiologen und Zahnärzte und wurde mit den DXAMessungen verglichen.

**Ergebnisse:**

Es wurden 57 Patienten mit DXA- und CT sowie 70 zufällig ausgewählte Patienten ohne Knochenerkrankungen als „Kontrollgruppe“ eingeschlossen. Die Messungen zeigten, dass der HPD mit dem Alter und der Knochendichte korreliert. Der mittlere HPD betrug 2,4 mm bei Knochengesunden, 0,9 mm bei Osteopenie, 0,8 mm bei Osteoporose und 5,3 mm bei Osteopetrose. Bei keinem Probanden der Kontrollgruppe lag der Wert unter 1 mm. Die Beziehung zwischen Knochendichte und HPD wurde bisher noch nicht beschrieben. Der HPD nimmt bei zunehmendem Alter, Osteopenie und Osteoporose ab. Bei Osteopetrose konnten wir einen Anstieg des HPD nachweisen.

**Schlussfolgerungen:**

HPD korreliert mit systemischen Knochenerkrankungen. Wir schlagen den HPD als neues Kriterium für die Beurteilung in CT und Digitaler Volumentomographie (DVT) oder Kegelstrahl-Computertomographie (CBCT) vor. Ein Schwellenwert von 1,0 mm kann als Indikator für das Vorliegen einer Osteopenie oder Osteoporose dienen. Der HPD kann in der radiologischen Bildgebung im Sinne eines Zufallsbefundes bzw. in der Zahnmedizin als Screening auf Osteoporose gewertet werden und als eine Indikation für eine Osteoporose-Abklärung mittel DXA gelten.

A new criterion for osteoporosis based on existing computed tomography (CT) images of the facial bones, which was performed for other reasons (e.g., fractures, tumors, inflammation), is introduced. In this sense, it is an incidental finding on CT images and an indication for further osteoporosis-specific diagnostic testing (e.g., DXA [dual-energy x-ray absorptiometry]) and medical treatment.

## Introduction

Osteoporosis is a chronic, systemic skeletal disease and one of the most common diseases of older age [1, 2, 7, 8, 15]. Osteoporosis, a bone disease that leads to fractures and disability, often goes unnoticed until the condition leads to injury. But research shows it can affect the teeth in noticeable ways, including tooth loss and gum disease. For patients with implants, dentures, and bridges, weak bones may lead to looser-fitting replacements. “Unfortunately, certain medications for osteoporosis, bisphosphonate drugs, also can cause dental issues—something all doctors should be aware of when prescribing any medications When a medical office puts someone on a new medication, they should send them to a dentist. Many of them can cause dry mouth, which can cause decay” as explained by Kalter [10]. According to the World Health Organization (WHO) criteria, osteoporosis is present in 30% of postmenopausal women [8, 15] and more than 200 million people are affected worldwide [2].

Osteoporosis can be visualized by different radiological methods. While osteoporosis may be suspected with conventional X‑ray, definite diagnosis is typically based on quantitative methods such as dual X‑ray absorptiometry (DXA) or quantitative computed tomography (QCT; see Table 1). The definition of osteoporosis according to WHO is based on DXA, which is considered the standard method of osteoporosis diagnosis.

**Table 1:** Methods for detection and quantification of osteoporosis

Method	Findings	Limitations
X‑ray/non-quantitative CT [3]	Radiolucency	Changes visible starting at approx. 40% of loss of density
	*Spongiosa reduction*	
	*Decreased cortical thickness*	
	Trabeculation	
	Frame vertebrae	
	Fractures	
Dual X‑ray-absorptiometry (DXA)	Measurement of bone density at lumbar spine, radial bone and femoral bone	WHO definition only includes lumbar spine and femoral bone
	Quantitative assessment in g/cm^2^	
	T value: comparison with specific reference population	
	Z value: comparison with population of same age	
Quantitative CT (QCT)	Quantitative measurement of density at lumbar vertebrae 1–3	More sensitive than DXA
	Conversion with reference phantom	
Digital X‑ray radiogrammetry (DXR)	Measurement of the cortical thickness of the three middle metacarpal bones of the hand	Measurement only of peripheral bones
Barnett–Nordin metacarpal index	Measurement of cortex-to-shaft ratio in a femur and a metacarpal and measurement of biconcavity in the spine	–

Outside the suggested sites of diagnosis defined by WHO, the detection and grading of osteoporosis diagnosis have also been the subject of various studies in head-and-neck imaging and dental imaging [9, 11, 13, 14]. As compared with dedicated quantitative imaging for the diagnosis of osteoporosis, these imaging procedures are typically performed for reasons other than osteoporosis. In the clinical setting these procedures may, therefore, serve as a first indicator of osteoporosis triggering further diagnostic procedures. Conventional X‑rays of teeth, focal plane tomography, and digital volume tomography (DVT) have been applied in a study setting to answer the question of whether osteoporosis may be suspected based on the findings of these imaging procedures [5, 12]. In these studies, complex multistage measurements and calculations were carried out.

We are looking for a simpler method as have been suggested by the Barnett–Nordin metacarpal index. Over the past few years, the authors have noticed a substantial variation in the thickness of the hard palate with a subjective decrease in older patients and those with osteoporosis. It can be assumed that the maxillary bone and the hard palate are subject to the same structural and morphological changes in patients affected by osteoporosis as has been verified for other osseous structures. We hypothesized that the criterion of “decreasing cortical thickness” mentioned above may have a significant influence on the overall diameter in a flat bone like the hard palate. The aim of this study was, therefore, to further investigate whether the thickness of the hard palate may serve as an indicator of osteoporosis in patients undergoing imaging for other reasons. The maxillary bone and specifically the hard palate are clearly delineated as anatomical structures and are easy to find and measure on cross-sectional imaging including CT and DVT.

## Methods

### Database of the study

**Fig. 1:**
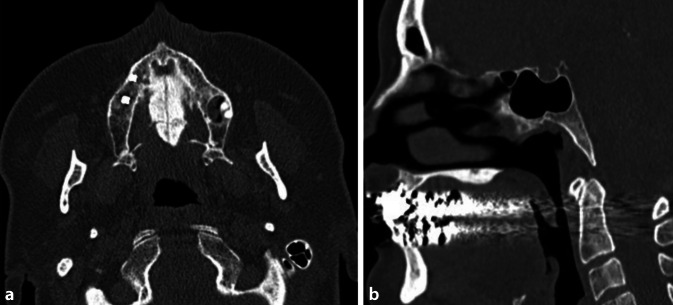
Example of an excluded patient. Exclusion was based on palate thickening due to torus palatinus. **a** Axial CT of bilobulated high-density masses from the midline of the hard palate (torus palatinus). **b** Shows the typical exostosis of torus palatinus in sagital view

This retrospective study was based on a radiology information system (RIS)/picture archiving and communication system (PACS) query. Patients were identified by an RIS-based, full-text search including all patients who underwent DXA and CT of the facial bones at our institution between 2008 and 2015. All patients identified were added to the “test group” if they did not meet any of the following exclusion criteria:

- Hard palate not in field of view,
- DXA at a time of more than 2 years from CT,
- Diseases or conditions affecting the maxilla, such as fractures, tumors in the area of measurement, diseases that may cause thickening (chronic inflammation, torus palatinus; see Fig. 1) or thinning (pressure atrophy in dentures, acute inflammation),
- Patients under osteoporosis therapy.

The classification of osteoporosis on DXA was based on the T value in clinical routine in all patients included in the test group:

- Normal bone density: T value up to −1 SD (standard deviation)
- Osteopenia: T value < −1 and > −2.5 SD
- Osteoporosis: T value < −2.5 SD

A “control group” consisted of randomly selected patients with polytrauma CT, no known bone disease, and without DXA. The control group was split into “control group 1” consisting of 50 patients younger than 40 years, and “control group 2” consisting of 20 patients older than 40 years. This study was approved by the local ethics committee (AZ: 302/17).

#### Measurement of thickness of the hard palate.

**Fig. 2:**
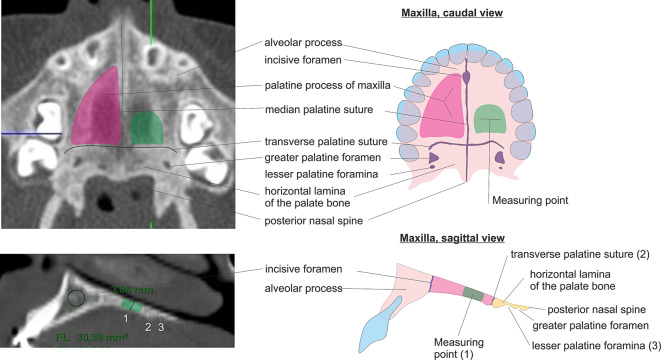
Definition of the measurement site on computed tomography images (*left*) and schematic overview of the anatomy (*right*)

At the beginning of our study, we defined a simple standard measurement for the thickness of hard palate as shown in Fig. 1. Measurements were performed on the left dorsal third of the palatine process of the maxilla. This site was chosen for the measurement because the contour of the hard palate runs horizontally for approx. 1 cm and may be measured easily (Fig. 2). All measurements were taken independently by a board-certified radiologist and a board-certified dentist at maximum magnification. All measurements and quantitative data from the DXA scan were transferred to Excel (Microsoft, USA) for statistical evaluation.

### Data analysis

Statistical analysis was performed using Microsoft 365 Excel, Version 2022, USA. Data are expressed as mean values and range (min, max), standard deviation (SD), median, and interquartile range (IQR). Scatter diagrams are used to show dependencies and correlations as well.

## Results

The patient population included a total of 449 patients with DXA and CT studies. Of these, we had to exclude 392 patients based on the exclusion criteria. We identified the remaining as the test group: 57 patients (mean age, 49.8).

We randomly selected patients without bone diseases from emergency room CTs without cranial injury to form the following control groups:

Control group 1: 50 people of age below 40 years (mean age 28.1 years)

Control group 2: 20 people of age over 40 years (mean age 62.9)

**Fig. 3:**
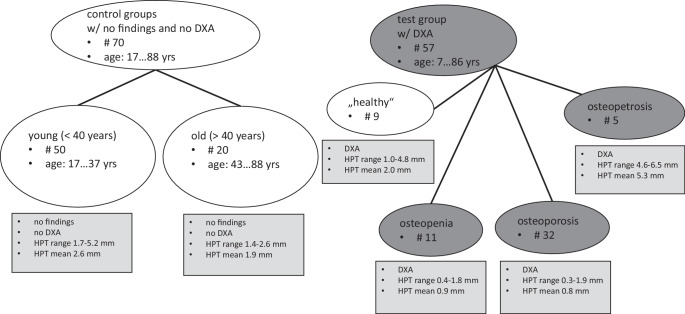
Overview of the groups, main characteristics, and results. *DXA* dual X‑ray absorptiometry, *HPT* hard palate thickness, *w/o* without, *yrs* years

For further results, see Fig. 3.

### Control groups

**Fig. 4:**
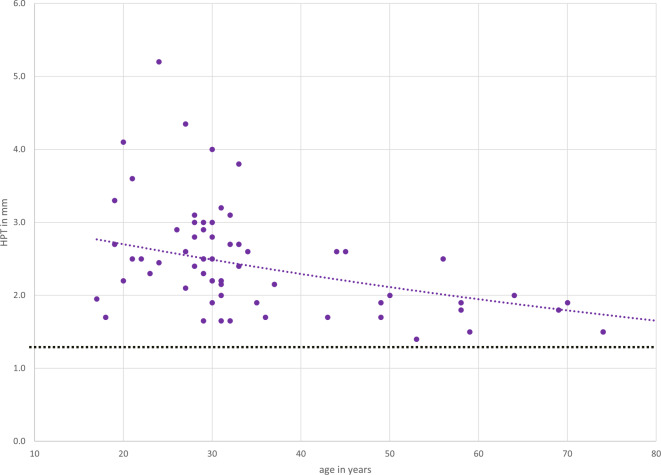
Decrease in hard palate thickness (*HPT*) with increasing age in healthy patients (control groups)

On average, a hard palate thickness (HPT) mean value of 2.6 mm was found in control group 1 (patients without findings, age < 40 years). None of the values in control group 1 fell below 1.7 mm (1.7–5.2 mm, SD = ±0.76, median = 2.5 mm, IQR = 0.81). The mean HPT in control group 2 (healthy patients > 40 years) was 1.9 mm (1.4–2.6 mm, SD = ±0.36, median = 1.85 mm, IQR = 0.4). The HPT was correlated with age, as shown in Fig. 4. Thus, HPT decreases with increasing age.

### Test group

**Fig. 5:**
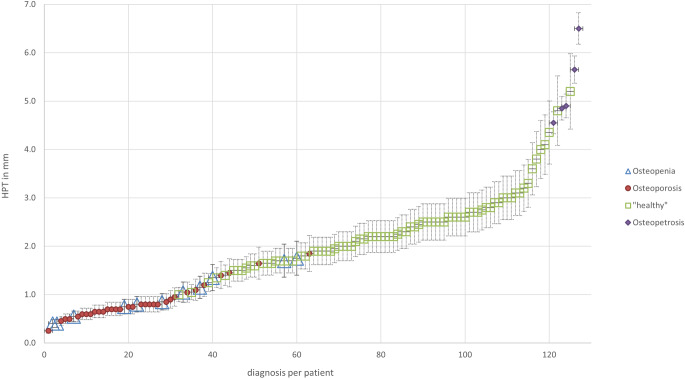
Relationship between hard palate thickness (*HPT*) and different bone diseases. In patients with osteopenia and osteoporosis HPT was lower, in patients with osteopetrosis it was substantially higher than in healthy controls (*green squares*)

Of 57 patients included we found 11 patients with osteopenia, 32 with osteoporosis, 5 with osteopetrosis, and 9 with normal findings on DXA (see Fig. 3). The mean HPT was 0.9 mm (0.4–1.8 mm, SD = ±0.47) in 11 patients with osteopenia, 0.8 mm (0.3–1.9 mm, SD = ±0.38) in 32 patients with osteoporosis, and 5.3 mm (4.6–6.5 mm, SD = ±0.82) in 5 with osteopetrosis. A correlation between HPT and decreased bone density in osteopenia/osteoporosis and in increased bone density (osteopetrosis) was established and is shown in Fig. 5.

### HPT in healthy and diseased patients

When summarizing results from the test group and the controls, the results for HPT were as follows:

- 2.4 mm (1.4–5.2) in 70 patients without systemic bone diseases (control groups 1 + 2).
- 0.9 mm (0.4–1.8 mm) in 11 patients with osteopenia
- 0.8 mm (0.3–1.9 mm) in 32 patients with osteoporosis
- 5.3 mm (4.6–6.5 mm) in 5 patients with osteopetrosis

**Fig. 6:**
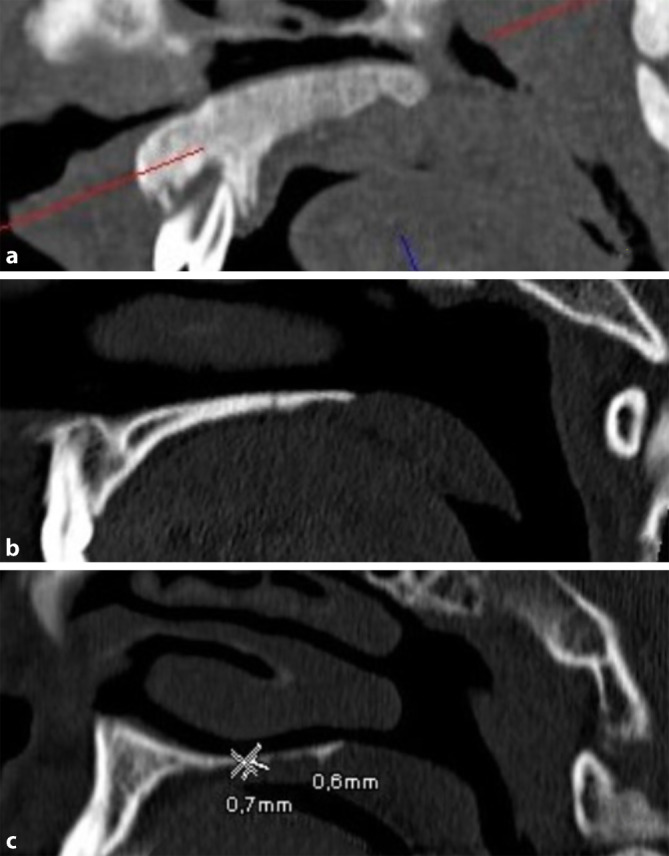
Example computed tomography images of hard palate thickness in patients with **a** osteopetrosis, **b** without bone disease, and **c** osteoporosis

None of the bone-healthy patients had values that fell below an HPT of 1.0 mm. Applying this lowest HPT of 1.0 mm from healthy patients as a potential threshold to the test group, e.g., as an additional finding, this would result in 21% of non-healthy patients being missed and 0% of healthy individuals would be referred for further clarification. An example of a measurement is shown in Fig. 6.

## Discussion

We propose HPT as a new CT criterion that may serve as an indicator of bone diseases such as osteopenia and osteoporosis in patients undergoing CT or DVT of the skull. To our knowledge, this is the first study to find a radiographic parameter for osteoporosis focusing on the hard palate in CT scans or dental tomosynthesis. In patients with an HPT of less than 1.0 mm as an incidental finding, further assessment for potential osteoporosis may be recommended.

The gold standard for diagnosing osteoporosis is DXA of the lumbar spine and hip, but it is not available in the emergency room or in an outpatient setting.

Previous studies have shown that cortical thickness of the femur, humerus, radius, and metacarpal bones can indicate osteoporotic changes [1, 17]. Others found a positive correlation between the bone mineral density (BMD) of the proximal humerus, using DXA and the cortical thickness of the proximal humeral diaphysis (Tingart measurement; [17]).

Spross et al. defined a deltoid tuberosity index to measure bone quality in patients with a proximal humerus fracture [16]. He et al. found a correlation between distal femoral cortical thickness, BMD, and T score [6].

Virtama et al. described the cortical indexes of the distal humerus and of the fingers as a simple and objective means of assessing one aspect of osteoporosis [18, 19]. All these previously suggested ways to assess for potential bone disease have one thing in common: They are rather complex and time-consuming.

We defined a simple and uniform measurement site in the dorsal third of the palatine process of the maxilla. This site is based on anatomical shape, which offers a reliable and reproducible measurement in this area. We found a correlation between HPT and decreased bone density in osteopenia/osteoporosis. Thus, by applying this simple measurement in the area of the hard palate, osteopenia/osteoporosis may be suspected. In addition, we found an increase in HPT in increased bone density in cases of osteopetrosis, which was expected and underlines the correlation between bone density and HPT.

The correlation of HPT with the DXA T value is not linear. A reliable differentiation between osteopenia and osteoporosis is not possible by applying HPT, at least based on the small patient population of this study cohort. Other authors also found that the cortical index cannot be considered a reliable estimate of the actual mineral content of bones [17]. However, this was not the aim of the study. The aim was to test for a potential initial criterion to hint at osteopenia and/or osteoporosis in patients undergoing CT or DVT of the skull for other reasons. The definitive diagnosis of osteopenia and/or osteoporosis will be made by DXA.

Measurements in patients without osteoporosis showed a correlation between HPT and age. Other studies also found a weak correlation between age and bone quality [6]. Bloom and Laws found a significantly lower combined cortical thickness of the humeral bone in patients older than 50 years of age [4, 17]. In our study the HPT of older patients did not decrease below the proposed threshold of 1 mm, showing HPT to be an osteoporosis indicator also in the cohort of older patients—the cohort in which osteoporosis is most prominent.

### Limitations

A limitation of the study was the exclusion of a larger number of patients based on a field of view lacking the hard palate. In many CTs of the skull, this area was not covered due to radiation protection reasons.

## Conclusion

The hard palate thickness (HPT) correlates with bone disease. We propose a new criterion for assessment on computed tomography (CT) and digital volume tomography (DVT) images. A threshold of 1.0 mm when applying a simple measurement of HPT on CT or DVT of the skull may serve as an indicator for potential osteopenia or osteoporosis. This simple measurement method could be used as screening tool, e.g., for dentists, based on existing CT or DVT images without any further imaging or treatment as the first step. In the case of an indication of osteoporosis, further diagnostic testing (e.g., DXA) and medical treatment are recommended.

## Data Availability

Data are available at the University Hospital Ulm, Diagnostic and Interventional Radiology, 89070 Ulm, Germany. Please contact the authors for access.

## Declarations

For this article no studies with human participants or animals were performed by any of the authors. All studies mentioned were in accordance with the ethical standards indicated in each case. This retrospective study was performed after consultation with the institutional ethics committee (The Ethics Committee of the University of Ulm reviewed and approved the study (AZ: 302/17)) and in accordance with national legal requirements.

The supplement containing this article is not sponsored by industry.
